# Multilocus sequence typing of *Cronobacter sakazakii *and *Cronobacter malonaticus *reveals stable clonal structures with clinical significance which do not correlate with biotypes

**DOI:** 10.1186/1471-2180-9-223

**Published:** 2009-10-23

**Authors:** Adam Baldwin, Michael Loughlin, Juncal Caubilla-Barron, Eva Kucerova, Georgina Manning, Christopher Dowson, Stephen Forsythe

**Affiliations:** 1 Biological Sciences Department, University of Warwick, Gibbet Hill Road, Coventry, CV4 7AL, UK; 2 School of Science and Technology, Nottingham Trent University, Clifton Lane, Nottingham, NG11 8NS, UK

## Abstract

**Background:**

The *Cronobacter *genus (*Enterobacter sakazakii*) has come to prominence due to its association with infant infections, and the ingestion of contaminated reconstituted infant formula. *C. sakazakii *and *C. malonaticus *are closely related, and are defined according their biotype. Due to the ubiquitous nature of the organism, and the high severity of infection for the immunocompromised, a multilocus sequence typing (MLST) scheme has been developed for the fast and reliable identification and discrimination of *C. sakazakii *and *C. malonaticus *strains. It was applied to 60 strains of *C. sakazakii *and 16 strains of *C. malonaticus*, including the index strains used to define the biotypes. The strains were from clinical and non-clinical sources between 1951 and 2008 in USA, Canada, Europe, New Zealand and the Far East.

**Results:**

This scheme uses 7 loci; *atp*D, *fus*A, *gln*S, *glt*B, *gyr*B, *inf*B, and *pps*. There were 12 sequence types (ST) identified in *C. sakazakii*, and 3 in *C. malonaticus*. A third (22/60) of *C. sakazakii *strains were in ST4, which had almost equal numbers of clinical and infant formula isolates from 1951 to 2008. ST8 may represent a particularly virulent grouping of *C. sakazakii *as 7/8 strains were clinical in origin which had been isolated between 1977 - 2006, from four countries. *C. malonaticus *divided into three STs. The previous *Cronobacter *biotyping scheme did not clearly correspond with STs nor with species.

**Conclusion:**

In conclusion, MLST is a more robust means of identifying and discriminating between *C. sakazakii *and *C. malonaticus *than biotyping. The MLST database for these organisms is available online at http://pubmlst.org/cronobacter/.

## Background

The genus *Cronobacter *is composed of Gram-negative, facultative anaerobic rods, which are members of the *Enterobacteriaceae *Family. It was formerly known as *Enterobacter sakazakii *and was divided into 15 biotypes [1]. The biotyping scheme was based on Voges-Proskauer, methyl red, indole, ornithine decarboxylase, motility, reduction of nitrate to nitrite, production of gas from D-glucose, malonate utilization and production of acid from myo-inositol and dulcitol. Based on 16S rDNA sequence analysis, we extended this further to 16 biotypes [2,3] which has contributed to the recent taxonomic revisions. Initially the *Cronobacter *genus was composed of 4 species; *C. sakazakii*, *C. turicensis*, *C. muytjensii*, *C. dublinensis*, plus a possible fifth species [4]. More recently, the species *C. malonaticus *sp. nov. was proposed [5]. This was initially regarded as a subspecies of *C. sakazakii *as the two species could not be distinguished according to 16S rDNA sequence analysis however DNA-DNA hybridisation studies revealed a <70% DNA relatedness. Consequently *C. sakazakii *consists of biotypes 1-4, 7 & 8, 11 & 13, and *C. malonaticus *contains biotypes 5, 9 and 14 [5].

*Cronobacter *spp. have come to prominence due to their association with infant infections, and cases linked to the ingestion of contaminated reconstituted infant formula [6-8]. However not all cases have been linked to formula ingestion. The organism is ubiquitous in the environment (water and soil) and food [9,10].

*Cronobacter *spp. cause infections across all age groups [11]. However neonates, particularly those of low-birth weight, are the major identified group at risk with a high mortality rate [6,11]. The organism is a rare cause of neonatal meningitis, necrotising enterocolitis (NEC) and sepsis. A number of outbreaks of *C. sakazakii *have been reported in neonatal intensive care units around the world [12-16]. The International Commission for Microbiological Specifications for Foods (2002) [17] has ranked *Cronobacter *spp. as 'severe hazard for restricted populations, life-threatening or substantial chronic sequelae or long duration'. The FAO/WHO [6,7,11] have undertaken three risk assessments of the organism in powdered infant formula, and the WHO [18] have published recommended procedures for the reconstitution of powdered infant formula to reduce the risk of infection to neonates. Together with the ubiquitous nature of the organism, and the high severity of infection for the immunocompromised, there is a need for a technique that enables fast and reliable classification and identification of *Cronobacter *strains worldwide.

Selected strains of *Cronobacter *spp. have been shown to invade human intestinal cells, replicate in macrophages, and invade the blood brain barrier [19,20]. Based on the clinical outcome of different pulsetypes during a neonatal intensive care unit outbreak it was proposed that certain types of *C. sakazakii *are particularly virulent [16,20]. Whether the virulence was linked to a particular genotype or phenotype warranted further investigation.

16S rDNA sequences can be useful to determine phylogenies between distantly related *Enterobacteriaeceae *[21]. However it is less discriminatory and unclear for more closely related organisms. An alternative to rDNA sequence analysis is the partial sequencing of protein-encoding genes. Additionally, for determining phylogenetic relationships, sequence data from more than one gene should be used to reduce the possibly of ambiguities caused by genetic recombination or specific selection [21,22]. A number of such genes have been used as phylogenetic markers for members of the *Enterobacteriaceae*. Genes which have been analysed include *rpoB*, *gyrB*, *mdh*, *inf*B and *recA *[23,24]. These results can be more reliable for species identification and determining intra- and inter-generic relationships than 16S rDNA gene sequencing. Recently, Kuhnert *et al. *[25] used three loci (*recN, rpoA *and *thdF*) for 30 species of *Enterobacteriaceae *including *Cronobacter *spp. Whereas our work is focussed on a higher resolution analysis of *C. sakazakii *and *C. malonaticus *using 7 loci. The genes under study were *atpD*, *fusA*, *glnS*, *gltB*, *gyrB*, *infB*, and *pps*. These genes encode for ATP synthase B subunit, elongation factor EF-2, glutamyl-tRNA synthetase, glutamate synthase large subunit, type IIA topoisomerase, initiation translation factor 2 and phosphoenol pyruvate synthase, respectively. These were compared with the previous biotyping scheme for *Cronobacter *spp. and also considered the source and isolation date of the strains.

## Results

### Development of the Cronobacter MLST scheme

Several criteria were used in the selection of all potential loci. Genes included were those encoding for putative housekeeping products necessary for biological roles in DNA repair, replication and amino acid biosynthesis. Genes that were either a) located near to or b) implicated as being, putative virulence factors and mobile elements were avoided as these may come under greater selective evolutionary pressures than other genes. The selected loci were distributed as much as possible across the chromosome to ensure that each locus was genetically unlinked. Each gene fragment was also required to be approximately 500 bp in length to facilitate the design of universal nested primers for each locus preferably in conserved flanking regions around a variable central core. Using these criteria, genes were selected and the chromosomal locations of all these loci were confirmed by bioinformatic analysis of the *C. sakazakii *BAA-894 genome sequence (Genbank accession number CP000785).

The genes selected for the MLST scheme (*atpD*, *fusA*, *glnS*, *gltB*, *gyrB*, *infB *and *pps*) are shown in Table 1 along with putative gene products, gene sizes, primers and location within the *C. sakazakii *ATCC BAA-894 genome.

**Table 1:** Oligonucleotide nested primer sequences for the amplification and sequencing of the seven loci from genes in *C. sakazakii *and *C. malonaticus*, with gene number and location of genes within the genome of the *C. sakazakii *strain ATCC BAA-894.

				Locus Primers (5'→3')	
				
Gene (Gene label)	Putative Gene Product	Chromosome location (bp)	Gene Size (bp)	Amplification	Sequencing
*atpD*	ATP synthase β chain	3,689,177 - 3,690,559	1,382	CGACATGAAAGGCGACAT	CGAAATGACCGACTCCAA
				
(ESA_04006)				TTAAAGCCACGGATGGTG	GGATGGCGATGATGTCTT

*fusA*	Elongation factor	3,275,843 - 3,277,957	2,114	GAAACCGTATGGCGTCAG	GCTGGATGCGGTAATTGA
				
(ESA_04401)				AGAACCGAAGTGCAGACG	CCCATACCAGCGATGATG

*glnS*	Glutaminyl-tRNA	660,368 - 662,035	1,667	GCATCTACCCGATGTACG	GGGTGCTGGATAACATCA
				
(ESA_02658)	synthetase			TTGGCACGCTGAACAGAC	CTTGTTGGCTTCTTCACG

*gltB*	Glutamate synthase	3,538,713 - 3,542,921	4,208	CATCTCGACCATCGCTTC	GCGAATACCACGCCTACA
				
(ESA_03606)	large subunit			CAGCACTTCCACCAGCTC	GCGTATTTCACGGAGGAG

*gyrB*	DNA gyrase B	3,719,848 - 3,722,262	2,414	TGCACCACATGGTATTCG	CTCGCGGGTCACTGTAAA
				
(ESA_03973)				CACCGGTCACAAACTCGT	ACGCCGATACCGTCTTTT

*infB*	Translation initiation	4,139,051 - 4,141,762	2,711	GAAGAAGCGGTAATGAGC	TGACCACGGTAAAACCTC
				
(ESA_03561)	factor IF-2			CGATACCACATTCCATGC	GGACCACGACCTTTATCC

*pps*	Phosphoenol-pyruvate	1,218,599 - 1,220,977	2,378	GTCCAACAATGGCTCGTC	ACCCTGACGAATTCTACG
				
(ESA_02102)	synthase			CAGACTCAGCCAGGTTTG	CAGATCCGGCATGGTATC

### Allelic variation

Novel sequence information for all seven loci was obtained from a collection of 60 *C. sakazakii *and 16 *C. malonaticus *strains. To assess the performance of the MLST scheme, *Cronobacter *strains were selected to be representative of the different biotypes (most of which were previously derived in an earlier study [3]), and were also distributed both temporally and geographically in terms of their isolation (See Additional file 1). *In silico *sequence data was also obtained for all the loci from *C. sakazakii *strain ATCC BAA-894 (Accession No. CP000785), *Citrobacter koseri *strain ATCC BAA-895 (Accession No. CP000822), and *Enterobacter *species strain 683 (Accession No. CP000653). The latter strain sequence data was used to root the data set. The seven alleles obtained for the *C. sakazakii *genome reference strain BAA-894 were identical to the online genome sequence (CP000785).

The mean allele length was 434 bp for the scheme and ranged between 363 bp (*glnS*) and 507 bp (*gltB*) in length (Table 2). All alleles within a particular locus were found to be of an identical length for all *Cronobacter *strains examined. Nucleotide sequence diversity at all seven loci is shown in Table 2. The proportion of variable sites varied from 10.8% (*atpD*) to 27.6% (*gyrB*) which extended over the whole section of the sequenced allele.

**Table 2:** Analysis of the seven MLST loci in the *Cronobacter *strains sampled.

Gene	Size (bp) of fragment analysed	No. of alleles	No. of polymorphic sites	Proportion of fragment as polymorphic sites (%)	*d*_*N*_/*d*_*S*_
*atpD*	390	12	42	10.8	0.006
*fusA*	438	12	69	15.8	0.061
*glnS*	363	12	72	19.8	0.062
*gltB*	507	11	118	23.3	0.059
*gyrB*	402	13	111	27.6	0.055
*infB*	441	12	87	19.7	0.079
*Pps*	495	15	123	24.8	0.033

Allele variation is not necessarily equally likely at every nucleotide of each locus. If a locus does not have a role affected by a selective pressure (such as antibiotic exposure) then nucleotide substitutions would frequently not be expected to change the amino acid sequence (synonymous) as changes are likely to be eliminated by purifying selection. By calculating the *d*_*N*_/*d*_*S *_ratio (non-synonymous substitutions to synonymous substitutions) the degree of selection operating on each locus can be estimated. The *d*_*N*_/*d*_*S *_ratio for all seven loci within *Cronobacter *strains was found to be significantly less than 1, ranging from 0.006 (*atpD*) to 0.079 (*infB*) (Table 2), indicating that no strong positive selective pressure was present at any of the loci selected, validating their suitability for inclusion in the MLST scheme.

### Assignment of allele and sequence types

The number of different alleles resolved from this *Cronobacter *MLST scheme at each locus ranged from 11 (*gltB*) to 15 (*pps*) alleles. The mean number of allele types per locus was found to be 13.4, providing the potential to distinguish >7 × 10^10 ^different genotypes and also making it highly unlikely to obtain identical sequence types (ST) by chance.

For the development of the MLST scheme, it was important to use a diverse collection of strains to obtain primarily different STs based upon known 16S rRNA gene sequencing and biotype to validate the scheme's effectiveness across the two *Cronobacter *species. For each unique allelic profile in the order *atpD, fusA*, *glnS*, *gltB, gyrB, infB *and *pps*, a unique ST was designated; See Additional file 1.

A total of 17 STs were found for the 78 strains examined (See Additional file 1); 12 STs for for *C. sakazakii *(n = 60), 3 *C. malonaticus *(n = 16), 1 *Cit. koseri *(n = 1) and 1 *Enterobacter *sp. 638 (n = 1). The sequences of each allele type at all seven loci, along with the allelic profiles and sequence types used for the multilocus sequence sequence analysis (MLSA) of the *Cronobacter *strains examined are available at http://pubmlst.org/cronobacter/.

The close genetic relationship between *C. sakazakii *and *C. malonaticus *was evident in that *atp*D allele 3 was identified both in *C. sakazakii *(ST3, ST17) and *C. malonaticus *(ST10). Apparently 'species specific' alleles were found across different STs e.g. the *Gln*S allele 3 was identified in *C. sakazakii *ST 3, 4,15 and 16, *fus*A allele 1 was in *C. sakazakii *ST1, 4, and 14, and three *C. malonaticus *STs had *fus*A allelic profile 7, and ST7 and ST10 had *glt*B allelic profile 7.

### Comparison of sequence type with source and biotype

In total 60 *C. sakazakii *and 16 *C. malonaticus *strains were analysed. Most strains analysed were associated with previous publications (See Additional file 1). The earliest isolate (NCIMB 8272) was from a can of dried milk powder, which was deposited in the culture collection in 1951, and the earliest clinical isolate (NCTC 9238) was deposited in 1953 [1].

*C. sakazakii *ST1 contained infant formula isolates from 1988-2003 from Russia, Netherlands, USA and UK. It included the ATCC BAA-894 strain from the Tennesse NICU outbreak [13] which has been sequenced (Accession number CP000785). Two strains were from milk powder and faeces. There were no known clinical outbreak isolates in ST1. *C. sakazakii *ST14 was a single strain from infant formula in France (1994) [16]. This ST varied by just a single nucleotide polymorphism from ST1 with respect to the *pps *locus. *C. sakazakii *ST3 strains were from infant formula, follow up formula, weaning food, and neonatal enteral feeding tubes. The strains were from 1988-2008, and were isolated in the Netherlands, UK, and Korea. There were no known clinical isolates, however there is no information available about the source for *C. sakazakii *strain ATCC 12868 in the culture collection.

*C. sakazakii *ST4 was the major (22/60) sequence type among the isolates. It contained almost equal numbers of clinical (n = 9) and infant formula (n = 7) isolates. This ST also included the Betty Hobbs 1951 isolate from a can of dried milk (NCIMB 8272) [1]. In contrast, strains in *C. sakazakii *ST8 were predominantly (7/8) clinical isolates from USA, Canada, and Czech Republic. The isolation dates ranged from 1977-2003, and included the *C. sakazakii *type strain (NCTC 11467^T^). The remaining strain was isolated in 2006 from infant formula in France. *C. sakazakii *ST12 included 5 strains from UK, USA, France and Czech Republic, at least 3 of which were clinical in origin.

*C. malonaticus *ST7 contained 11 strains which were primarily clinical in origin from the Czech Republic, isolated between 1977 and 2004. *C. malonaticus *ST11 contained 3 clinical strains from the Czech Republic, biotypes 2a, 14a, and 13b which were isolated in 1983 [30]. *C. malonaticus *ST10 was composed of two strains from chinese herbs which were both isolated in 2005.

Biotypes did not always correspond with sequence types or *Cronobacter *species (See Additional file 1). For example, biotype 2 was primarily distributed over *C. sakazakii *ST1 and 3, with two other strains in ST4. The index strain for biotype 2a was in *C. malonaticus *ST11, and a second strain was in *C. sakazakii *ST12. Biotype 1 was in *C. sakazakii *ST4, 8,13,15,17 and 18. *C. malonaticus *is defined as biotypes 5, 9 and 14 [5]. However biotype 5 was in *C. malonaticus *ST7 and 10, and *C. sakazakii *ST16. Biotype 9 was only in *C. malonaticus *ST7. Whereas biotype 14 and 14a were in *C. malonaticus *ST7, and ST11. Biotypes 2a, 4a, 13a, and 13b are conventionally assigned to *C. sakazakii *[3]. However, *C. malonaticus *ST7 included the index strains for biotypes 4a and 13a, and *C. malonaticus *ST11 included the index strains for biotypes 2a and 13b.

### Relationships of C. sakazakii, C. malonaticus, Cit. koseri and Enterobacter sp. 638 using concatenated nucleotide sequences

**Figure 1:**
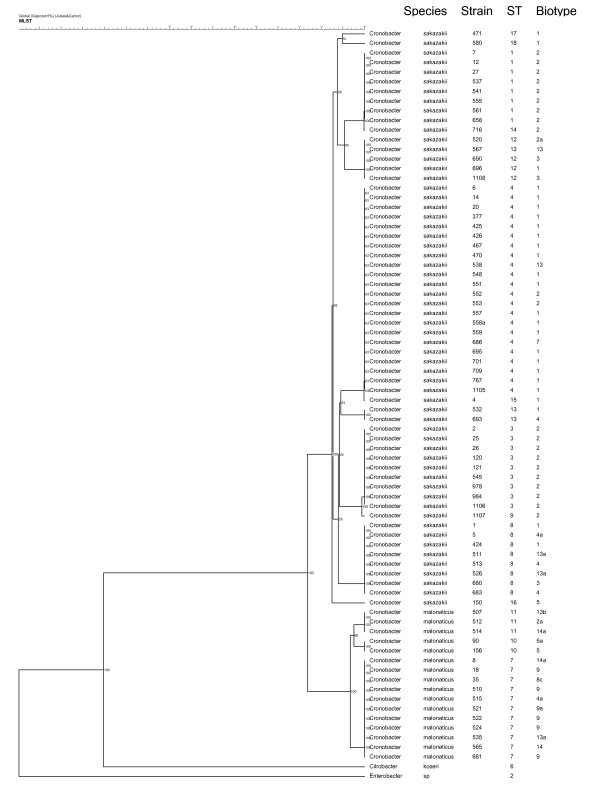
**Phylogenetic tree of concatenated nucleotide sequences from the seven loci, using the UPGMA method, Jukes-Cantor**. Bootstrap values are shown for 1,000 replicates.

In order to assess all the loci together in one tree, concatenated nucleotide sequences were used. Concatenated nucleotide sequences (3,036 bp) for the 15 *Cronobacter *STs, *Cit. koseri *and *Enterobacter *sp. 638 were analysed using the UPGMA method (Figure 1). The *Cronobacter *species were fully resolved, falling into distinctive clusters of strains. The *Cronobacter *species were clearly separated from the *Enterobacter *sp. strain 638 and also by a lesser extent from the *Cit. koseri *strain (100% bootstraps). *C. sakazakii *and *C. malonaticus *separated from each other at 2.6% divergence (100% bootstrap value), and from *Citrobacter koseri *at 13% divergence. Figure 1 also shows the distribution of biotypes across the sequence types.

### Analysis of recombination among C. sakazakii

Bacteria existing as clonal populations evolve diversity by the accumulation of point mutations, while non-clonal populations evolve more through recombination within or between species. In this study identical alleles were found within species and between the two *Cronobacter *species (See Additional file 1).

Evidence for clonal or recombining populations can be estimated by assessing the level of linkage between alleles at different loci around the chromosome. The index of association (*I*_*A*_) [34] measures the extent of linkage. An *I*_*A *_not significantly greater than zero after 1,000 computer randomizations would suggest that a single species population (monophyletic) is in linkage equilibrium (freely recombining), while a population with an *I*_*A *_significantly greater than zero (p < 0.001) is considered to be in linkage disequilibrium (clonal). *C. sakazakii *examined had an *I*_*A *_value of 0.28 (p value < 0.01) and therefore indicates a more clonal that freely recombining population. Further analysis will be undertaken as part of a subsequent study, along with other *Cronobacter *spp..

## Discussion and Conclusion

The diversity of *Enterobacter sakazakii *was well acknowledged prior to the taxonomic revision to the *Cronobacter *genus, which was based on DNA-DNA hybridisation, 16S rDNA sequence analysis, and biotyping [5]. The earlier biotyping scheme was extremely useful in aiding the definition of the various *Cronobacter *species, especially due to the close genetic relationship of *C. sakazakii *and *C. malonaticus *which initially was regarded as a subspecies of *C. sakazakii *[4].

Nevertheless, phenotyping is in part subjective, and a DNA based scheme is preferred for its robustness. This study has used 7 loci for a MLST scheme for *C. sakazakii *and *C. malonaticus*. Strains were chosen to represent the diversity of *C. sakazakii *and *C. malonaticus *based on biotype, geographic and temporal distribution, and source (environmental, formula, clinical). The strains were from Europe, USA, Canada, Russia, New Zealand, Korea and China. The isolation dates ranged over 57 years from 1951 to 2008.

As MLST uses multiple loci, a greater degree of variation and better resolution for MLSA and for inferring evolutionary and epidemiological relatedness can be obtained than by a single locus alone. Twelve sequence types of *C. sakazakii *were assigned. ST4 contained the largest number of strains, both clinical, infant formula, and milk powder isolates, from USA, Canada, Europe and Russia. The earliest isolate dates from 1951 and demonstrates the ubiquity of this sequence type. Many (18/22) of these strains were biotype 1, which was previously shown to be the most numerous biotype (60/189) [3].

Previously Caubilla-Barron et al. [16] and Townsend et al. [20] reported on *C. sakazakii *infections in neonatal intensive care unit outbreak, which involved 4 pulsetypes. Only one pulsetype (PT2) was associated with all the deaths and therefore indicated that *C. sakazakii *strains may vary in their virulence potential. PT2 strains caused necrotizing enterocolitis (NEC), septicaemia, and meningitis. These strains were all in ST4. Other strains, associated with non-fatal NEC, neonatal colonisation, and infant formulas were in ST12, 13 and 14.

ST8 is of particular interest as 7/8 strains were clinical in origin, the eighth isolate being isolated from infant formula. These organisms isolated between 1977 and 2006 were from USA, Canada, France and the Czech Republic. ST8 also contains the *C. sakazakii *type strain (NCTC 11467^T^, equivalent ATCC 29544^T^) and interestingly the index strains for biotypes 1, 3 and 4. Some of these strains have previously been studied by Pagotto et al. [33] and Postupa and Aldovα [35]. ST(8) therefore merits further investigation, as it may represent a particularly virulent type of *C. sakazakii *strains.

Similarly ST7 in *C. malonaticus *was dominated (8/11) by clinical isolates, however this grouping may be biased as 5 clinical isolates (510, 515, 521, 522, 524) were epidemiologically linked. There is also a predominance of biotype 9 in this sequence type, which may in part explain why that biotype was previously associated with clinical source; 10/13 strains [3].

The MLST scheme is openly available on the internet for other workers and will assist in the identification and discrimination of *C. sakazakii *and *C. malonaticus *based on DNA sequence in place of the far less reliable biotyping approach, which in isolation is essentially of no phylogenetic value and little epidemiological value. The role of biotyping in the identification and discrimination of *C. sakazakii *and *C. malonaticus *needs to be seriously reviewed. Even within the sample of isolates examined MLSA has already identified 1 or 2 STs which appear to be associated with enhanced virulence, and this may aid our understanding of the pathogenicity of this ubiquitous organism.

## Methods

### Source of strains and biotyping

Strains were chosen on the basis of their species, biotype, geographic and temporal distribution, source and clinical outcome (See Additional file 1). This included the type strains *C. sakazakii *NCTC 11467^T^, and *C. malonaticus *CDC 1058-77^T^, biotype index strains, infant formula and clinical isolates, from Europe, USA, Canada, Russia, New Zealand, Korea and China, ranging from 1951 to 2008. The majority of these have associated published articles (See Additional file 1). Biotyping was as according to Iversen et al. [3].

### DNA isolation and PCR

Genomic DNA was prepared using GenElute™ Bacterial Genomic DNA Kit (Sigma) and 1.5 ml of overnight culture grown in TSB broth as per the manufacturer's instructions.

### Selection of MLST gene loci

MLST loci were selected by comparing genome sequence data for *C. sakazakii *(strain ATCC BAA-894; http://genome.wustl.edu), *Cit. koseri *(strain ATCC BAA-895; http://genome.wustl.edu) and *Enterobacter *sp. strain 638 http://www.jgi.doe.gov/ using the Artemis Comparison Tool (ACT) and the Double ACT program available at http://www.sanger.ac.uk/Software/ACT/ and http://www.hpa-bioinfotools.org.uk/pise/double_act.html, respectively.

### Primer design

Amplification and nested sequencing primers for the MLST loci were then designed to conserved areas of these genes using Primer3 available at http://frodo.wi.mit.edu/[36].

For each locus, primers were designed to have a similar melting temperature and were found to successfully amplify by PCR a panel of *C. sakazakii *and *C. malonaticus *strains (Table 1 and Additional file 1). Reaction conditions for all the primers were as follows: initial denaturation at 94°C for 2 min; 30 cycles of denaturation at 94°C for 1 min, primer annealing at 58°C for 1 min, extension at 72°C for 2 min; followed by a final extension step of 72°C for 5 min. Each 50 μl amplification reaction mixture comprised ~10 ng chromosomal DNA, 10 μl Q solution (Qiagen, Crawley, UK), 20 pmol forward and reverse primer, 1× PCR buffer (Qiagen) containing 1.5 mM MgCl_2_, 0.8 mM deoxynucleotide triphosphates and 1.25 U Taq (Qiagen). The amplification product was then purified using MinElute UF plates (Qiagen) following the manufacturer's protocol before being used in a sequencing reaction.

### Multilocus sequence analysis

Using the nested sequencing primers, nucleotide sequences were determined at least once on each DNA strand with BigDye Terminator Ready Reaction Mix v3.1 (PE Biosystems, Foster City, US) under standard sequencing conditions according to the manufacturer's protocol. Unincorporated dye terminators were removed by precipitation with 95% alcohol. The reaction products were separated and detected on an ABI PRISM genetic analyser 3100 (PE Biosystems) using a standard sequencing module with a Performance Optimised Polymer and 5 cm array. The sequences from both strands of a given locus of the same isolate were aligned, trimmed to the desired length and edited using SeqMan II (DNA Star software, Madison, US).

### Allele and Sequence Type designation

Arbitrary allelic numbers were assigned to each unique allele for a given locus. After sequencing and assigning allele types to all seven loci each isolate was then designated by a combination of seven numbers called an allelic profile that represented a sequence type (ST) for that particular isolate (eg. ST1). A novel sequence type (ST) designation was given to each isolate with a unique allelic profile while subsequent isolates with an identical allelic profile were assigned the same ST identifier and considered to be isogenic strains as they were indistinguishable at all seven loci. All alleles within the MLST scheme were in frame, to aid with analysis.

### Linkage analysis

Linkage analysis was carried out by using the index of association (*I*_*A*_), as defined previously [37]. We examined whether alleles were randomly associated, that is, at linkage equilibrium, indicating a freely recombining population, or non-randomly associated, that is, at linkage disequilibrium, implying a clonal population structure. If there is linkage equilibrium, i.e., a random association between alleles of different loci, *I*_*A *_= 0. If *I*_*A *_is significantly different from 0, it indicates that recombination has been rare or absent and that the population has a clonal structure [34].

## Authors' contributions

AB designed and carried out the MLST, assisted by EK. JC, ML, GM and CD provided technical expertise. Thanks to Edward Hurrell for additional strain biotyping. AB and SF wrote the manuscript. SF managed the project. All authors read and approved the final manuscript.

## Supplementary Material

Additional file 1 **MLST analysis of the *Cronobacter *isolates showing their source, geographic location and species**. The data provided shows the spacial, temporal and source of strains used in this study, and reference where the strains have been used in previous publications. Click here for file
