# Supplementary material for: Multilocus sequence typing of Cronobacter sakazakii and Cronobacter malonaticus reveals stable clonal structures with clinical significance which do not correlate with biotypes
Source: BMC Microbiol. 2009 Oct 23;9:223. doi: 10.1186/1471-2180-9-223 (PMC2770063; doi:10.1186/1471-2180-9-223)
Supplement: Additional file 1 — MLST analysis of the Cronobacter isolates showing their source, geographic location and species. The data provided shows the spacial, temporal and source of strains used in this study, and reference where the strains have been used in previous publications. [file 1471-2180-9-223-S1.DOC]

Addtional file 1. MLST analysis of the *Cronobacter* isolates showing their source, geographic location and species.

| **ST** | **Isolate** | **Other identifier** | **Farmer biotype** | **Country** | **Source** | **Year** | **References** | **Allelic profile** | | | | | | |
| --- | --- | --- | --- | --- | --- | --- | --- | --- | --- | --- | --- | --- | --- | --- |
|  |  |  |  |  |  |  |  | ***atpD*** | ***fusA*** | ***glnS*** | ***gltB*** | ***gyrB*** | ***infB*** | ***pps*** |
| *C. sakazakii* | | | | | | | | | | | | | | |
| 1 | 537 |  | 2 | Russia | Infant formula | 1988 | [26] | 1 | 1 | 1 | 1 | 1 | 1 | 1 |
| 1 | 541 |  | 2 | Netherlands | Infant formula | 1988 | [26] | 1 | 1 | 1 | 1 | 1 | 1 | 1 |
| 1 | 555 |  | 2 | Netherlands | Infant formula | 1988 | [26] | 1 | 1 | 1 | 1 | 1 | 1 | 1 |
| 1 | 561 |  | 2 | Netherlands | Infant formula | 1988 | [26] | 1 | 1 | 1 | 1 | 1 | 1 | 1 |
| 1 | 658 | BAA-894 | 2 | USA | Infant formula | 2001 | [13,32] | 1 | 1 | 1 | 1 | 1 | 1 | 1 |
| 1 | 7 |  | 2 | UK | Infant formula | 2003 | [2,31] | 1 | 1 | 1 | 1 | 1 | 1 | 1 |
| 1 | 27 |  | 2 | UK | Milk powder | 2004 |  | 1 | 1 | 1 | 1 | 1 | 1 | 1 |
| 1 | 12 |  | 2* | Czech Rep. | Faeces | 2004 | [1,3] | 1 | 1 | 1 | 1 | 1 | 1 | 1 |
| 3 | 545 |  | 2 | Netherlands | Infant formula | 1988 | [26] | 3 | 3 | 3 | 5 | 3 | 3 | 3 |
| 3 | 978 |  | 2 | UK | EFT^a^ | 2007 | [28] | 3 | 3 | 3 | 5 | 3 | 3 | 3 |
| 3 | 984 |  | 2 | UK | EFT | 2007 | [28] | 3 | 3 | 3 | 5 | 3 | 3 | 3 |
| 3 | 25 |  | 2 | Korea | FUF^b^ | 2004 | [2,31] | 3 | 3 | 3 | 5 | 3 | 3 | 3 |
| 3 | 26 |  | 2 | Korea | FUF | 2004 |  | 3 | 3 | 3 | 5 | 3 | 3 | 3 |
| 3 | 120 |  | 2 | Korea | FUF | 2005 |  | 3 | 3 | 3 | 5 | 3 | 3 | 3 |
| 3 | 121 |  | 2 | Korea | FUF | 2005 |  | 3 | 3 | 3 | 5 | 3 | 3 | 3 |
| 3 | 1106 |  | 2 | UK | Weaning food | 2008 | [29] | 3 | 3 | 3 | 5 | 3 | 3 | 3 |
| 3 | 2 | ATCC 12868 | 2 | Unknown | Unknown | Unknown | [2,19,31,32] | 3 | 3 | 3 | 5 | 3 | 3 | 3 |
| 4 | 553 |  | 2 | Netherlands | Clinical | 1983 | [27] | 5 | 1 | 3 | 3 | 5 | 5 | 4 |
| 4 | 557 |  | 1 | Netherlands | Clinical | 1983 | [27] | 5 | 1 | 3 | 3 | 5 | 5 | 4 |
| 4 | 558 |  | 1 | Netherlands | Clinical | 1983 | [27] | 5 | 1 | 3 | 3 | 5 | 5 | 4 |
| 4 | 695 |  | 1 | France | Clinical | 1994 | [16,20] | 5 | 1 | 3 | 3 | 5 | 5 | 4 |
| 4 | 701 |  | 1 | France | Clinical | 1994 | [16,20] | 5 | 1 | 3 | 3 | 5 | 5 | 4 |
| 4 | 709 |  | 1 | France | Clinical | 1994 | [16,20] | 5 | 1 | 3 | 3 | 5 | 5 | 4 |
| 4 | 767 |  | 1 | France | Clinical | 1994 | [16,20] | 5 | 1 | 3 | 3 | 5 | 5 | 4 |
| 4 | 6 | LB | 1 | Canada | Clinical | 2003 | [33] | 5 | 1 | 3 | 3 | 5 | 5 | 4 |
| 4 | 20 |  | 1 | Czech Rep. | Clinical | 2004 | [2] | 5 | 1 | 3 | 3 | 5 | 5 | 4 |
| 4 | 538 |  | 13 | Russia | Infant formula | 1988 | [26] | 5 | 1 | 3 | 3 | 5 | 5 | 4 |
| 4 | 548 |  | 1 | Germany | Infant formula | 1988 | [26] | 5 | 1 | 3 | 3 | 5 | 5 | 4 |
| 4 | 552 |  | 2 | Netherlands | Infant formula | 1988 | [26] | 5 | 1 | 3 | 3 | 5 | 5 | 4 |
| 4 | 14 |  | 1 | UK | Infant formula | 2003 |  | 5 | 1 | 3 | 3 | 5 | 5 | 4 |
| 4 | 425 |  | 1 | France | Infant formula | 2006 |  | 5 | 1 | 3 | 3 | 5 | 5 | 4 |
| 4 | 426 |  | 1 | France | Infant formula | 2006 |  | 5 | 1 | 3 | 3 | 5 | 5 | 4 |
| 4 | 467 |  | 1 | France | Infant formula | 2006 |  | 5 | 1 | 3 | 3 | 5 | 5 | 4 |
| 4 | 377 | NCIMB 8272 | 1 | UK | Milk powder | 1951 | [1,2] | 5 | 1 | 3 | 3 | 5 | 5 | 4 |
| 4 | 1105 |  | 1 | UK | Weaning food | 2008 | [29] | 5 | 1 | 3 | 3 | 5 | 5 | 4 |
| 4 | 551 |  | 1 | Netherlands | Environmental | 1988 | [26] | 5 | 1 | 3 | 3 | 5 | 5 | 4 |
| 4 | 559 |  | 1 | Netherlands | Wash brush | 1988 | [26] | 5 | 1 | 3 | 3 | 5 | 5 | 4 |
| 4 | 470 | ATCC 29004 | 1 | USA | Unknown | Unknown | [2] | 5 | 1 | 3 | 3 | 5 | 5 | 4 |
| 4 | 686 | CDC 9369-75 | 7* | USA | Unknown | 1975 | [1,3] | 5 | 1 | 3 | 3 | 5 | 5 | 4 |
| 8 | 680 | CDC 996-77 | 3* | USA | Clinical | 1977 | [1,3] | 11 | 8 | 7 | 5 | 8 | 15 | 10 |
| 8 | 683 |  | 4* | USA | Clinical | 1977 | [1,3] | 11 | 8 | 7 | 5 | 8 | 15 | 10 |
| 8 | 1 | NCTC 11467^T^ | 1* | USA | Clinical | 1980 | [1,2, 3,5,31,32] | 11 | 8 | 7 | 5 | 8 | 15 | 10 |
| 8 | 511 |  | 13a | Czech Rep. | Clinical | 1983 | [30] | 11 | 8 | 7 | 5 | 8 | 15 | 10 |
| 8 | 513 |  | 4 | Czech Rep. | Clinical | 1983 | [30] | 11 | 8 | 7 | 5 | 8 | 15 | 10 |
| 8 | 526 |  | 13a | Czech Rep. | Clinical | 1983 | [30] | 11 | 8 | 7 | 5 | 8 | 15 | 10 |
| 8 | 5 | LA | 4a | Canada | Clinical | 2003 | [33] | 11 | 8 | 7 | 5 | 8 | 15 | 10 |
| 8 | 424 |  | 1 | France | Infant formula | 2006 |  | 11 | 8 | 7 | 5 | 8 | 15 | 10 |
| 9 | 1107 |  | 2 | UK | Weaning food | 2008 | [29] | 21 | 10 | 9 | 5 | 3 | 3 | 3 |
| 12 | 520 |  | 2a | Czech Rep. | Clinical | 1983 | [30] | 18 | 17 | 10 | 12 | 18 | 24 | 18 |
| 12 | 690 |  | 3 | France | Clinical | 1994 | [16] | 18 | 17 | 10 | 12 | 18 | 24 | 18 |
| 12 | 696 |  | 1 | France | Clinical | 1994 | [16,20] | 18 | 17 | 10 | 12 | 18 | 24 | 18 |
| 12 | 1108 |  | 3 | UK | Weaning food | 2008 | [29] | 18 | 17 | 10 | 12 | 18 | 24 | 18 |
| 12 | 567 | CDC 9363-73 | 13* | USA | Unknown | 1980 | [1,3] | 18 | 17 | 10 | 12 | 18 | 24 | 18 |
| 13 | 693 |  | 4 | France | Clinical | 1994 | [16] | 15 | 14 | 15 | 13 | 22 | 5 | 16 |
| 13 | 532 |  | 1 | Germany | Infant formula | 1988 | [26] | 15 | 14 | 15 | 13 | 22 | 5 | 16 |
| 14 | 716 |  | 2 | France | Infant formula | 1994 | [16,20] | 1 | 1 | 1 | 1 | 1 | 1 | 11 |
| 15 | 4 | SK90 | 1 | Canada | Clinical | 2003 | [31,33] | 5 | 9 | 3 | 3 | 5 | 5 | 4 |
| 16 | 150 |  | 5 | Korea | Spices | 2005 |  | 15 | 1 | 3 | 9 | 14 | 19 | 15 |
| 17 | 471 |  | 1 | Korea | Infant formula | 2006 |  | 3 | 12 | 16 | 5 | 16 | 20 | 14 |
| 18 | 580 | NCTC 9238 | 1 | UK | Clinical | 1953 | [1] | 20 | 18 | 16 | 10 | 3 | 20 | 20 |
| *C. malonaticus* | | | | | | | | | | | | | | |
| 7 | 681 | CDC 1058-77^T^ | 9* | USA | Clinical | 1977 | [1,3,5] | 10 | 7 | 6 | 7 | 9 | 14 | 9 |
| 7 | 510 |  | 9 | Czech Rep. | Clinical | 1983 | [30] | 10 | 7 | 6 | 7 | 9 | 14 | 9 |
| 7 | 515 |  | 4a* | Czech Rep. | Clinical | 1983 | [3,30] | 10 | 7 | 6 | 7 | 9 | 14 | 9 |
| 7 | 524 |  | 9 | Czech Rep. | Clinical | 1983 | [30] | 10 | 7 | 6 | 7 | 9 | 14 | 9 |
| 7 | 521 |  | 9a* | Czech Rep. | Clinical | 1983 | [3,30] | 10 | 7 | 6 | 7 | 9 | 14 | 9 |
| 7 | 522 |  | 9 | Czech Rep. | Clinical | 1983 | [30] | 10 | 7 | 6 | 7 | 9 | 14 | 9 |
| 7 | 18 |  | 9 | Czech Rep. | Clinical | 2004 |  | 10 | 7 | 6 | 7 | 9 | 14 | 9 |
| 7 | 35 |  | 8c | USA | Clinical | 2005 |  | 10 | 7 | 6 | 7 | 9 | 14 | 9 |
| 7 | 535 |  | 13a* | New Zealand | Milk powder | 1988 | [3,26] | 10 | 7 | 6 | 7 | 9 | 14 | 9 |
| 7 | 8 |  | 14a | Czech Rep. | Weaning food | 2004 | [2] | 10 | 7 | 6 | 7 | 9 | 14 | 9 |
| 7 | 565 | CDC 1895-33 | 14* | USA | Faeces | 1980 | [1,3] | 10 | 7 | 6 | 7 | 9 | 14 | 9 |
| 10 | 90 |  | 5a | China | Herbs | 2005 | [2] | 3 | 7 | 11 | 7 | 10 | 16 | 8 |
| 10 | 156 |  | 5 | China | Herbs | 2005 |  | 3 | 7 | 11 | 7 | 10 | 16 | 8 |
| 11 | 507 |  | 13b* | Czech Rep. | Clinical | 1983 | [1,3,30] | 17 | 7 | 17 | 11 | 17 | 22 | 12 |
| 11 | 512 |  | 2a* | Czech Rep. | Clinical | 1983 | [1,3,30] | 17 | 7 | 17 | 11 | 17 | 22 | 12 |
| 11 | 514 |  | 14a | Czech Rep. | Clinical | 1983 | [1,30] | 17 | 7 | 17 | 11 | 17 | 22 | 12 |
| *Citrobacter koseri* | | | | | | | | | | | | | | |
| 6 | BAA-895 |  | NA |  | Clinical |  |  | 9 | 6 | 5 | 6 | 7 | 13 | 7 |
| *Enterobacter* sp. | | | | | | | | | | | | | | |
| 2 | 638 |  | NA |  | Environmental |  |  | 2 | 2 | 2 | 2 | 2 | 2 | 2 |

* Biotype index strain [3]. ^T^ Species type strain. ^a^ Enteral feeding tube. ^b^ Follow up formula.
